# Bis(2-cyclo­butyl­imino­methyl-4,6-dihydro­seleno­phenolato)zinc(II)

**DOI:** 10.1107/S1600536809031596

**Published:** 2009-08-15

**Authors:** Di Zhu, Lei Lei, Xi-Bin Dai, Qing-Fu Zeng

**Affiliations:** a Engineering Research Center for Clean Production of Textile Dyeing and Printing, Ministry of Education, Wuhan 430073, People’s Republic of China

## Abstract

In the title complex, [Zn(C_11_H_12_NOSe_2_)_2_], the Zn^II^ atom is four-coordinated by two *O*,*N*-bidentate Schiff base ligands in a distorted tetra­hedral geometry.

## Related literature

For background to Schiff bases, see: Shi *et al.* (2008 ▶); Xu *et al.* (2009 ▶). For reference structural data, see: Allen *et al.* (1987 ▶).
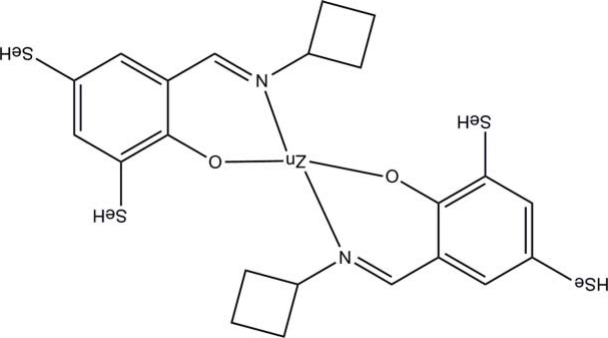


## Experimental

### 

#### Crystal data

[Zn(C_11_H_12_NOSe_2_)_2_]
*M* *_r_* = 729.64
Triclinic, 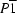

*a* = 8.0876 (6) Å
*b* = 12.2986 (16) Å
*c* = 12.7956 (16) Å
α = 93.166 (6)°
β = 108.216 (6)°
γ = 95.716 (6)°
*V* = 1197.9 (2) Å^3^
*Z* = 2
Mo *K*α radiation
μ = 7.12 mm^−1^
*T* = 296 K
0.32 × 0.28 × 0.25 mm

#### Data collection

Enraf–Nonius CAD-4 diffractometer
Absorption correction: ψ scan (North *et al.*, 1968) *T*_min_ = 0.209, *T*_max_ = 0.269 (expected range = 0.131–0.168)
6184 measured reflections
4183 independent reflections
3165 reflections with *I* > 2σ(*I*)
*R*_int_ = 0.024
3 standard reflections every 200 reflections intensity decay: 1%

#### Refinement

*R*[*F* ^2^ > 2σ(*F* ^2^)] = 0.046
*wR*(*F* ^2^) = 0.130
*S* = 1.05
4183 reflections
284 parameters
H-atom parameters constrained
Δρ_max_ = 0.73 e Å^−3^
Δρ_min_ = −1.48 e Å^−3^

### 

Data collection: *CAD-4 Software* (Enraf–Nonius, 1989 ▶); cell refinement: *CAD-4 Software*; data reduction: *XCAD4* (Harms & Wocadlo, 1995 ▶); program(s) used to solve structure: *SHELXS97* (Sheldrick, 2008 ▶); program(s) used to refine structure: *SHELXL97* (Sheldrick, 2008 ▶); molecular graphics: *SHELXTL* (Sheldrick, 2008 ▶); software used to prepare material for publication: *SHELXTL*.

## Supplementary Material

Crystal structure: contains datablocks global, I. DOI: 10.1107/S1600536809031596/hb5040sup1.cif

Structure factors: contains datablocks I. DOI: 10.1107/S1600536809031596/hb5040Isup2.hkl

Additional supplementary materials:  crystallographic information; 3D view; checkCIF report

## Figures and Tables

**Table 1:** Selected bond lengths (Å)

Zn1—N1	2.002 (5)
Zn1—N2	1.987 (5)
Zn1—O1	1.908 (4)
Zn1—O2	1.911 (4)

**Fig. 1.**
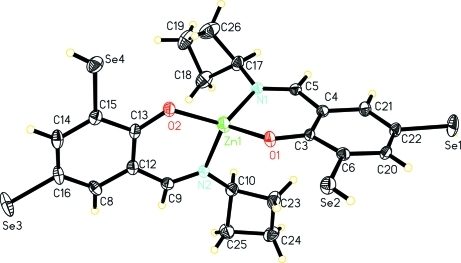
The molecular structure of (I) showing 30% probability displacement ellipsoids.

## supplementary crystallographic information

### Comment

There has been much research interest in Schiff base metal complexes due to their molecular architectures and biological activities (Shi *et al.*, 2008; Xu *et al.*, 2009). In this work, we report here the crystal structure of the title compound, (I). In (I), all bond lengths are within normal ranges (Allen *et al.*, 1987) (Fig. 1). The Zn(II) is four-coordinated in a distorted tetrahedral coordination by two N atoms and two O atoms of the Schiff base ligands.

### Experimental

A mixture of 3,5-dihydroseleno-2-hydroxybenzaldehyde (564 mg, 2 mmol), cyclobutanamine (142 mg, 2 mmol) and ZnCl_2_ (1 mmol, 134 mg) in methanol (10 ml) was stirred for 1 h. After keeping the filtrate in air for 8 d, colorless blocks of (I) were formed.

### Refinement

All H atoms were positioned geometrically (C—H = 0.93–0.96 Å, Se—H = 0.82Å) and refined as riding, with *U*_iso_(H) = 1.2*U*_eq_(carrier).

### Crystal data

**Table:**

[Zn(C_11_H_12_NOSe_2_)_2_]	*Z* = 2
*M**_r_* = 729.64	*F*(000) = 704
Triclinic, *P*1	*D*_x_ = 2.023 Mg m^−^^3^
Hall symbol: -P 1	Mo *K*α radiation, λ = 0.71073 Å
*a* = 8.0876 (6) Å	Cell parameters from 25 reflections
*b* = 12.2986 (16) Å	θ = 9–12°
*c* = 12.7956 (16) Å	µ = 7.12 mm^−^^1^
α = 93.166 (6)°	*T* = 296 K
β = 108.216 (6)°	Block, colorless
γ = 95.716 (6)°	0.32 × 0.28 × 0.25 mm
*V* = 1197.9 (2) Å^3^	

### Data collection

**Table:**

Enraf–Nonius CAD-4 diffractometer	3165 reflections with *I* > 2σ(*I*)
Radiation source: fine-focus sealed tube	*R*_int_ = 0.024
graphite	θ_max_ = 25.0°, θ_min_ = 1.7°
ω/2θ scans	*h* = −9→9
Absorption correction: ψ scan (North *et al.*, 1968)	*k* = −11→14
*T*_min_ = 0.209, *T*_max_ = 0.269	*l* = −15→14
6184 measured reflections	3 standard reflections every 200 reflections
4183 independent reflections	intensity decay: 1%

### Refinement

**Table:**

Refinement on *F*^2^	Primary atom site location: structure-invariant direct methods
Least-squares matrix: full	Secondary atom site location: difference Fourier map
*R*[*F*^2^ > 2σ(*F*^2^)] = 0.046	Hydrogen site location: inferred from neighbouring sites
*wR*(*F*^2^) = 0.130	H-atom parameters constrained
*S* = 1.05	*w* = 1/[σ^2^(*F*_o_^2^) + (0.0591*P*)^2^ + 4.4413*P*] where *P* = (*F*_o_^2^ + 2*F*_c_^2^)/3
4183 reflections	(Δ/σ)_max_ < 0.001
284 parameters	Δρ_max_ = 0.73 e Å^−^^3^
0 restraints	Δρ_min_ = −1.48 e Å^−^^3^

### Special details

**Table:**

Geometry. All e.s.d.'s (except the e.s.d. in the dihedral angle between two l.s. planes) are estimated using the full covariance matrix. The cell e.s.d.'s are taken into account individually in the estimation of e.s.d.'s in distances, angles and torsion angles; correlations between e.s.d.'s in cell parameters are only used when they are defined by crystal symmetry. An approximate (isotropic) treatment of cell e.s.d.'s is used for estimating e.s.d.'s involving l.s. planes.
Refinement. Refinement of *F*^2^ against ALL reflections. The weighted *R*-factor *wR* and goodness of fit *S* are based on *F*^2^, conventional *R*-factors *R* are based on *F*, with *F* set to zero for negative *F*^2^. The threshold expression of *F*^2^ > σ(*F*^2^) is used only for calculating *R*-factors(gt) *etc*. and is not relevant to the choice of reflections for refinement. *R*-factors based on *F*^2^ are statistically about twice as large as those based on *F*, and *R*- factors based on ALL data will be even larger.

### Fractional atomic coordinates and isotropic or equivalent isotropic displacement parameters (Å^2^)

**Table:**

	*x*	*y*	*z*	*U*_iso_*/*U*_eq_	
C3	−0.0178 (8)	0.4782 (5)	0.1563 (5)	0.0251 (13)	
C4	−0.1460 (8)	0.5305 (5)	0.0777 (5)	0.0270 (14)	
C5	−0.1629 (8)	0.6448 (5)	0.0877 (5)	0.0275 (14)	
H5	−0.2448	0.6703	0.0285	0.033*	
C6	−0.0144 (8)	0.3665 (5)	0.1286 (5)	0.0285 (14)	
C8	0.2692 (9)	0.8003 (5)	0.6800 (5)	0.0334 (15)	
H8	0.1939	0.7846	0.7206	0.040*	
C9	0.0370 (9)	0.7100 (5)	0.5214 (5)	0.0333 (15)	
H9	−0.0288	0.7034	0.5692	0.040*	
C10	−0.2221 (9)	0.6202 (6)	0.3895 (6)	0.0351 (16)	
H10	−0.2957	0.6743	0.3521	0.042*	
C12	0.2138 (8)	0.7668 (5)	0.5674 (5)	0.0316 (15)	
C13	0.3264 (8)	0.7890 (5)	0.5038 (5)	0.0301 (15)	
C14	0.5457 (9)	0.8801 (6)	0.6732 (6)	0.0381 (17)	
H14	0.6567	0.9177	0.7088	0.046*	
C15	0.4932 (8)	0.8481 (5)	0.5614 (6)	0.0323 (15)	
C16	0.4309 (9)	0.8554 (6)	0.7316 (6)	0.0394 (17)	
C17	−0.1131 (9)	0.8311 (5)	0.1571 (6)	0.0381 (17)	
H17	−0.2085	0.8365	0.0888	0.046*	
C18	−0.1400 (11)	0.8977 (6)	0.2528 (8)	0.053 (2)	
H18A	−0.2618	0.9052	0.2436	0.063*	
H18B	−0.0817	0.8737	0.3246	0.063*	
C19	−0.0413 (11)	0.9963 (6)	0.2242 (8)	0.059 (2)	
H19A	0.0410	1.0404	0.2879	0.071*	
H19B	−0.1158	1.0415	0.1747	0.071*	
C20	−0.1314 (8)	0.3059 (5)	0.0389 (5)	0.0298 (14)	
H20	−0.1264	0.2313	0.0265	0.036*	
C21	−0.2633 (8)	0.4682 (5)	−0.0173 (5)	0.0314 (15)	
H21	−0.3447	0.5025	−0.0692	0.038*	
C22	−0.2581 (8)	0.3579 (5)	−0.0336 (5)	0.0313 (15)	
C23	−0.2772 (10)	0.5116 (6)	0.3183 (7)	0.049 (2)	
H23A	−0.1871	0.4629	0.3319	0.059*	
H23B	−0.3285	0.5187	0.2400	0.059*	
C24	−0.4130 (10)	0.4865 (7)	0.3787 (7)	0.048 (2)	
H24A	−0.5283	0.5064	0.3405	0.058*	
H24B	−0.4207	0.4121	0.3998	0.058*	
C25	−0.3008 (10)	0.5711 (7)	0.4725 (6)	0.048 (2)	
H25A	−0.3676	0.6196	0.5008	0.058*	
H25B	−0.2175	0.5401	0.5320	0.058*	
C26	0.0420 (13)	0.9166 (6)	0.1673 (9)	0.066 (3)	
H26A	0.1535	0.8983	0.2142	0.079*	
H26B	0.0501	0.9379	0.0970	0.079*	
N1	−0.0797 (7)	0.7162 (4)	0.1679 (4)	0.0288 (12)	
N2	−0.0372 (7)	0.6683 (4)	0.4224 (4)	0.0297 (12)	
O1	0.0927 (6)	0.5283 (4)	0.2458 (4)	0.0331 (11)	
O2	0.2880 (6)	0.7612 (4)	0.3987 (4)	0.0372 (11)	
Se1	−0.41129 (11)	0.27481 (7)	−0.16339 (7)	0.0561 (3)	
H1	−0.5133	0.2804	−0.1676	0.084*	
Se2	0.16624 (10)	0.29622 (6)	0.22268 (6)	0.0406 (2)	
H2	0.1451	0.2297	0.2068	0.061*	
Se3	0.50639 (12)	0.89915 (10)	0.88567 (7)	0.0660 (3)	
H3	0.4248	0.9208	0.9027	0.099*	
Se4	0.64728 (10)	0.88355 (6)	0.48096 (7)	0.0435 (2)	
H4	0.7125	0.9397	0.5105	0.065*	
Zn1	0.08132 (11)	0.67103 (7)	0.30778 (7)	0.0407 (2)	

### Atomic displacement parameters (Å^2^)

**Table:**

	*U*^11^	*U*^22^	*U*^33^	*U*^12^	*U*^13^	*U*^23^
C3	0.025 (3)	0.026 (3)	0.022 (3)	−0.005 (3)	0.008 (3)	−0.002 (3)
C4	0.024 (3)	0.031 (3)	0.027 (3)	−0.002 (3)	0.010 (3)	0.001 (3)
C5	0.028 (3)	0.026 (3)	0.026 (3)	0.002 (3)	0.007 (3)	0.004 (3)
C6	0.028 (3)	0.032 (4)	0.027 (3)	0.002 (3)	0.012 (3)	0.002 (3)
C8	0.038 (4)	0.041 (4)	0.023 (3)	0.011 (3)	0.011 (3)	0.000 (3)
C9	0.036 (4)	0.037 (4)	0.029 (4)	−0.002 (3)	0.015 (3)	0.001 (3)
C10	0.028 (4)	0.039 (4)	0.035 (4)	−0.002 (3)	0.008 (3)	0.001 (3)
C12	0.029 (3)	0.031 (4)	0.030 (4)	0.003 (3)	0.004 (3)	−0.002 (3)
C13	0.029 (3)	0.026 (3)	0.030 (4)	0.002 (3)	0.003 (3)	−0.004 (3)
C14	0.028 (4)	0.040 (4)	0.037 (4)	0.008 (3)	−0.003 (3)	−0.009 (3)
C15	0.028 (3)	0.029 (4)	0.037 (4)	0.003 (3)	0.006 (3)	0.000 (3)
C16	0.033 (4)	0.046 (4)	0.029 (4)	0.006 (3)	−0.002 (3)	−0.012 (3)
C17	0.043 (4)	0.023 (3)	0.039 (4)	0.004 (3)	0.002 (3)	0.002 (3)
C18	0.050 (5)	0.035 (4)	0.076 (6)	0.009 (4)	0.023 (4)	0.002 (4)
C19	0.046 (5)	0.035 (4)	0.090 (7)	0.002 (4)	0.018 (5)	−0.009 (4)
C20	0.033 (4)	0.022 (3)	0.032 (4)	−0.005 (3)	0.012 (3)	−0.003 (3)
C21	0.026 (3)	0.035 (4)	0.030 (4)	0.002 (3)	0.004 (3)	−0.002 (3)
C22	0.026 (3)	0.032 (4)	0.030 (4)	−0.007 (3)	0.005 (3)	−0.005 (3)
C23	0.043 (5)	0.048 (5)	0.055 (5)	−0.011 (4)	0.021 (4)	−0.014 (4)
C24	0.039 (4)	0.047 (5)	0.060 (5)	−0.009 (3)	0.023 (4)	0.002 (4)
C25	0.037 (4)	0.066 (5)	0.041 (4)	−0.010 (4)	0.019 (3)	0.000 (4)
C26	0.076 (7)	0.038 (5)	0.100 (8)	0.006 (4)	0.052 (6)	0.008 (5)
N1	0.033 (3)	0.024 (3)	0.028 (3)	0.002 (2)	0.007 (2)	0.004 (2)
N2	0.031 (3)	0.030 (3)	0.025 (3)	−0.003 (2)	0.007 (2)	−0.002 (2)
O1	0.034 (3)	0.033 (3)	0.026 (2)	0.004 (2)	0.003 (2)	−0.005 (2)
O2	0.035 (3)	0.043 (3)	0.030 (3)	−0.009 (2)	0.011 (2)	−0.006 (2)
Se1	0.0466 (5)	0.0481 (5)	0.0492 (5)	−0.0046 (4)	−0.0123 (4)	−0.0193 (4)
Se2	0.0428 (4)	0.0318 (4)	0.0409 (4)	0.0097 (3)	0.0028 (3)	0.0024 (3)
Se3	0.0512 (5)	0.1048 (8)	0.0278 (4)	0.0108 (5)	−0.0035 (4)	−0.0202 (4)
Se4	0.0340 (4)	0.0438 (5)	0.0533 (5)	−0.0036 (3)	0.0181 (3)	0.0005 (4)
Zn1	0.0417 (5)	0.0415 (5)	0.0346 (5)	0.0002 (4)	0.0088 (4)	−0.0034 (4)

### Geometric parameters (Å, °)

**Table:**

C3—O1	1.290 (7)	C17—H17	0.9800
C3—C6	1.405 (9)	C18—C19	1.509 (12)
C3—C4	1.432 (9)	C18—H18A	0.9700
C4—C21	1.413 (9)	C18—H18B	0.9700
C4—C5	1.429 (9)	C19—C26	1.518 (12)
C5—N1	1.276 (8)	C19—H19A	0.9700
C5—H5	0.9300	C19—H19B	0.9700
C6—C20	1.361 (9)	C20—C22	1.386 (9)
C6—Se2	1.896 (6)	C20—H20	0.9300
C8—C16	1.356 (10)	C21—C22	1.368 (9)
C8—C12	1.393 (9)	C21—H21	0.9300
C8—H8	0.9300	C22—Se1	1.900 (6)
C9—N2	1.272 (8)	C23—C24	1.545 (10)
C9—C12	1.452 (9)	C23—H23A	0.9700
C9—H9	0.9300	C23—H23B	0.9700
C10—N2	1.472 (8)	C24—C25	1.530 (11)
C10—C23	1.518 (10)	C24—H24A	0.9700
C10—C25	1.522 (10)	C24—H24B	0.9700
C10—H10	0.9800	C25—H25A	0.9700
C12—C13	1.416 (9)	C25—H25B	0.9700
C13—O2	1.299 (8)	C26—H26A	0.9700
C13—C15	1.420 (9)	C26—H26B	0.9700
C14—C15	1.380 (9)	Zn1—N1	2.002 (5)
C14—C16	1.384 (11)	Zn1—N2	1.987 (5)
C14—H14	0.9300	Zn1—O1	1.908 (4)
C15—Se4	1.883 (7)	Zn1—O2	1.911 (4)
C16—Se3	1.902 (7)	Se1—H1	0.8200
C17—N1	1.470 (8)	Se2—H2	0.8200
C17—C18	1.519 (11)	Se3—H3	0.8200
C17—C26	1.522 (11)	Se4—H4	0.8200
			
O1—C3—C6	120.5 (6)	C18—C19—H19B	114.1
O1—C3—C4	123.8 (6)	C26—C19—H19B	114.1
C6—C3—C4	115.6 (5)	H19A—C19—H19B	111.3
C21—C4—C5	116.3 (6)	C6—C20—C22	118.6 (6)
C21—C4—C3	119.7 (6)	C6—C20—H20	120.7
C5—C4—C3	124.0 (6)	C22—C20—H20	120.7
N1—C5—C4	128.0 (6)	C22—C21—C4	120.6 (6)
N1—C5—H5	116.0	C22—C21—H21	119.7
C4—C5—H5	116.0	C4—C21—H21	119.7
C20—C6—C3	124.4 (6)	C21—C22—C20	120.9 (6)
C20—C6—Se2	118.2 (5)	C21—C22—Se1	120.6 (5)
C3—C6—Se2	117.5 (5)	C20—C22—Se1	118.3 (5)
C16—C8—C12	121.3 (7)	C10—C23—C24	86.9 (6)
C16—C8—H8	119.3	C10—C23—H23A	114.2
C12—C8—H8	119.3	C24—C23—H23A	114.2
N2—C9—C12	126.8 (6)	C10—C23—H23B	114.2
N2—C9—H9	116.6	C24—C23—H23B	114.2
C12—C9—H9	116.6	H23A—C23—H23B	111.3
N2—C10—C23	118.6 (6)	C25—C24—C23	88.1 (5)
N2—C10—C25	121.4 (6)	C25—C24—H24A	114.0
C23—C10—C25	89.4 (6)	C23—C24—H24A	114.0
N2—C10—H10	108.6	C25—C24—H24B	114.0
C23—C10—H10	108.6	C23—C24—H24B	114.0
C25—C10—H10	108.6	H24A—C24—H24B	111.2
C8—C12—C13	120.5 (6)	C10—C25—C24	87.4 (6)
C8—C12—C9	116.2 (6)	C10—C25—H25A	114.1
C13—C12—C9	123.3 (6)	C24—C25—H25A	114.1
O2—C13—C12	125.4 (6)	C10—C25—H25B	114.1
O2—C13—C15	118.6 (6)	C24—C25—H25B	114.1
C12—C13—C15	116.0 (6)	H25A—C25—H25B	111.3
C15—C14—C16	119.1 (6)	C19—C26—C17	88.0 (6)
C15—C14—H14	120.5	C19—C26—H26A	114.0
C16—C14—H14	120.5	C17—C26—H26A	114.0
C14—C15—C13	122.6 (6)	C19—C26—H26B	114.0
C14—C15—Se4	119.3 (5)	C17—C26—H26B	114.0
C13—C15—Se4	118.1 (5)	H26A—C26—H26B	111.2
C8—C16—C14	120.6 (6)	C5—N1—C17	118.3 (5)
C8—C16—Se3	121.0 (6)	C5—N1—Zn1	120.5 (4)
C14—C16—Se3	118.4 (5)	C17—N1—Zn1	121.0 (4)
N1—C17—C18	119.7 (6)	C9—N2—C10	118.7 (6)
N1—C17—C26	117.8 (6)	C9—N2—Zn1	123.1 (5)
C18—C17—C26	87.0 (6)	C10—N2—Zn1	118.2 (4)
N1—C17—H17	110.1	C3—O1—Zn1	125.6 (4)
C18—C17—H17	110.1	C13—O2—Zn1	125.8 (4)
C26—C17—H17	110.1	C22—Se1—H1	109.5
C19—C18—C17	88.5 (7)	C6—Se2—H2	109.5
C19—C18—H18A	113.9	C16—Se3—H3	109.5
C17—C18—H18A	113.9	C15—Se4—H4	109.5
C19—C18—H18B	113.9	O1—Zn1—O2	121.3 (2)
C17—C18—H18B	113.9	O1—Zn1—N2	113.3 (2)
H18A—C18—H18B	111.1	O2—Zn1—N2	94.9 (2)
C18—C19—C26	87.5 (6)	O1—Zn1—N1	94.9 (2)
C18—C19—H19A	114.1	O2—Zn1—N1	123.5 (2)
C26—C19—H19A	114.1	N2—Zn1—N1	109.6 (2)
